# Blue Light Treatment but Not Green Light Treatment After Pre-exposure to UV-B Stabilizes Flavonoid Glycoside Changes and Corresponding Biological Effects in Three Different Brassicaceae Sprouts

**DOI:** 10.3389/fpls.2020.611247

**Published:** 2021-01-28

**Authors:** Susanne Neugart, Petra Majer, Monika Schreiner, Éva Hideg

**Affiliations:** ^1^Division Quality and Sensory of Plant Products, Georg-August-Universität Göttingen, Göttingen, Germany; ^2^Institute of Plant Biology, Biological Research Centre, Hungarian Academy of Sciences, Szeged, Hungary; ^3^Leibniz Institute of Vegetable and Ornamental Crops e.V., Grossbeeren, Germany; ^4^Department of Plant Biology, University of Pécs, Pécs, Hungary

**Keywords:** LEDs (light emitting diode), kale, UV-radiation, kohlrabi, rocket salad

## Abstract

Ultraviolet-B (UV-B; 280–315 nm) radiation induces the biosynthesis of secondary plant metabolites such as flavonoids. Flavonoids could also be enhanced by blue (420–490 nm) or green (490–585 nm) light. Flavonoids act as antioxidants and shielding components in the plant’s response to UV-B exposure. They are shown to quench singlet oxygen and to be reactive to hydroxyl radical. The aim was to determine whether treatment with blue or green light can alter flavonoid profiles after pre-exposure to UV-B and whether they cause corresponding biological effects in Brassicaceae sprouts. Based on their different flavonoid profiles, three vegetables from the Brassicaceae were selected. Sprouts were treated with five subsequent doses (equals 5 days) of moderate UV-B (0.23 kJ m^–2^ day^–1^ UV-B_BE_), which was followed with two subsequent (equals 2 days) doses of either blue (99 μmol m^–2^ s^–1^) or green (119 μmol m^–2^ s^–1^) light. In sprouts of kale, kohlrabi, and rocket salad, flavonoid glycosides were identified by HPLC-DAD-ESI-MS^n^. Both *Brassica oleracea* species, kale and kohlrabi, showed mainly acylated quercetin and kaempferol glycosides. In contrast, in rocket salad, the main flavonol glycosides were quercetin glycosides. Blue light treatment after the UV-B treatment showed that quercetin and kaempferol glycosides were increased in the *B. oleracea* species kale and kohlrabi while—contrary to this—in rocket salad, there were only quercetin glycosides increased. Blue light treatment in general stabilized the enhanced concentrations of flavonoid glycosides while green treatment did not have this effect. Blue light treatment following the UV-B exposure resulted in a trend of increased singlet oxygen scavenging for kale and rocket. The hydroxyl radical scavenging capacity was independent from the light quality except for kale where an exposure with UV-B followed by a blue light treatment led to a higher hydroxyl radical scavenging capacity. These results underline the importance of different light qualities for the biosynthesis of reactive oxygen species that intercept secondary plant metabolites, but also show a pronounced species-dependent reaction, which is of special interest for growers.

## Introduction

Plants have photosensory mechanisms to detect ultraviolet-B (UV-B; 280–315 nm) radiation (Heijde and Ulm, 2012; Jenkins, 2014) and therefore protect and repair sensitive targets from direct and indirect UV-induced injury (Britt, 1996; Jansen and Bornman, 2012). One line of defense is the increased biosynthesis of flavonoids and hydroxycinnamic acids (Jansen et al., 2008; Neugart and Schreiner, 2018). However, the impact of moderate UV-B, below ecologically summer day doses, during cultivation is less investigated (Schreiner et al., 2012; Neugart and Schreiner, 2018). Changes in the biosynthesis of flavonoid glycosides and hydroxycinnamic acid derivatives in response to UV-B depends on the chemical structure of the compounds (Olsson et al., 1998; Neugart et al., 2012a, 2014; Harbaum-Piayda et al., 2016). Light-emitting diodes (LEDs) are the most innovative light sources and create new possibilities for indoor cultivation. Blue and red LEDs were the first choice for manufacturers as these wavelengths are efficiently absorbed by chlorophylls (Kaiser et al., 2019). It is a widespread but erroneous belief that plants simply prefer blue and red light and poorly absorb green light (Smith et al., 2017). Less than 50% of green light (500–600 nm range) is reflected by plant chloroplasts, and green light reaches deeper leaf tissues more efficiently than blue or red wavelengths, which are shielded with chlorophyll-containing layers (Terashima et al., 2009). It was shown recently that flavonoid biosynthesis can be enhanced by blue light (420–490 nm) or green light (490–585 nm), but species differ in their response. While blue light treatment (30–100 μmol m^–2^ s^–1^) of lettuce resulted in higher total phenols (Johkan et al., 2010; Samuoliene et al., 2017), higher doses of blue light within the light spectrum (200 μmol m^–2^ s^–1^) resulted in a reduction of total phenolics in lamb’s lettuce (Wojciechowska et al., 2015). For green light treatment (30 μmol m^–2^ s^–1^) of lettuce, an increase of total phenolics was reported by the same authors. While UV-B radiation activates the UVR8 photoreceptor, blue and partly green light trigger the cryptochromes among other blue light receptors (Fuglevand et al., 1996; Heijde and Ulm, 2012; Jenkins, 2014; Tissot and Ulm, 2020). Extraordinarily, the light signaling of these photoreceptors, both UVR8 and cryptochromes, is mediated through COP1 operating as signaling center (Lau and Deng, 2012). While indoor farming offers the chance to tailor make light spectra in the visible spectrum, UV LEDs are not commonly used due to higher costs. One idea to overcome this issue is to have UV bulbs for the UV-B treatment and later on use visible light to stabilize or further increase the UV effects. There are no information how structurally different flavonol glycosides and hydroxycinnamic acids respond to different light qualities after pre-exposure with UV-B. Therefore, some basic principles need to be investigated for the later development of recipes.

The effect of light conditions during growth on flavonoids is of special importance, because these compounds act as antioxidants and shielding components in the plant’s response to UV-B exposure (Edreva, 2005; Agati et al., 2013; Waterman et al., 2017) and are well-known antioxidants in human nutrition (Pan et al., 2010). Flavonoids are structurally diverse polyphenols that are ubiquitous in plants where they naturally occur as glycosides. *Brassica* vegetable leaves have high concentrations of flavonoids and hydroxycinnamic acids (Neugart et al., 2012a). The main flavonoids of Brassicaceae are quercetin and kaempferol glycosides. In kale, kaempferol glycosides show different antioxidant activities dependent on their chemical structure (Fiol et al., 2012). Rocket salad is known for high concentrations of quercetin glycosides (Martínez-Sánchez et al., 2007).

Flavonoids, as antioxidants, can quench singlet oxygen *in vivo* (Triantaphylides and Havaux, 2009) and are also reactive to other reactive oxygen species (ROS) such as hydroxyl radicals (Husain et al., 1987). Glycosylation reduces the antioxidant activity of flavonoids and affects the accumulation, stability, and solubility of flavonoids (Gachon et al., 2005; Bowles et al., 2006). The intracellular accumulation at sites of ROS production underlines the important antioxidant properties of flavonoids (Hernandez et al., 2009). Remarkably, it was previously shown that hydroxycinnamic acids act as scavengers of ROS induced by UV-B radiation (Edreva, 2005). Many studies investigated the overall antioxidant activity (such as TEAC) among different *Brassica* vegetables (Podsedek, 2007) or focus on total phenolic content and specific radical scavenging capacities (Li et al., 2011). Nevertheless, there are less scavenging information on plant-specific ROS such as singlet oxygen and hydroxyl radical and their correlation to flavonoid glycosides.

The ability of plants to accumulate protective UV-absorbing compounds such as flavonoids in response to days or weeks of exposure to UV is well known (Searles et al., 2001). It was shown that epidermal flavonoids are more important in response to UV-A and UV-B than mesophyll flavonoids and even epidermal hydroxycinnamic acids (Burchard et al., 2000; Kolb et al., 2001). However, it was also shown that flavonoids can absorb light in the lower wavelength region of the visible spectrum, e.g., blue light (Gitelson et al., 2017), which can result in higher flavonoid concentrations (Matysiak and Kowalski, 2019). In addition, the absorption spectra of flavonoids depend on their chemical structure (Stochmal and Oleszek, 2007). Nevertheless, flavonoids such as rutin (absorption maxima at 260 and 360 nm) and hydroxycinnamic acids such as chlorogenic acid (absorption maximum at 320 nm) are relevant UV-absorbing compounds (Solovchenko and Merzlyak, 2008). How specific complex flavonoid glycosides and hydroxycinnamic acids contribute to the absorption capacity is still largely unclear, but will support non-destructive measurements of flavonoids in the future.

We hypothesize that (i) UV-B pre-exposure leads to an increase in flavonoid glycosides and hydroxycinnamic acids with high antioxidant activity, (ii) blue and green light after UV-B pre-exposure contributes to obtain the higher concentrations of flavonoid glycosides and hydroxycinnamic acids, (iii) these light-induced changes in flavonoid and hydroxycinnamic acid profiles result in an increased ROS scavenging capacity and increased absorption ability, and (iv) there are specific species within the Brassicaceae family. This knowledge will be transferable to recipes for indoor use and subsequent storage conditions, as it is important to increase not only the yield but also phenolic compounds that can act as color, flavor, and health-promoting compounds.

## Materials and Methods

**FIGURE 1:**
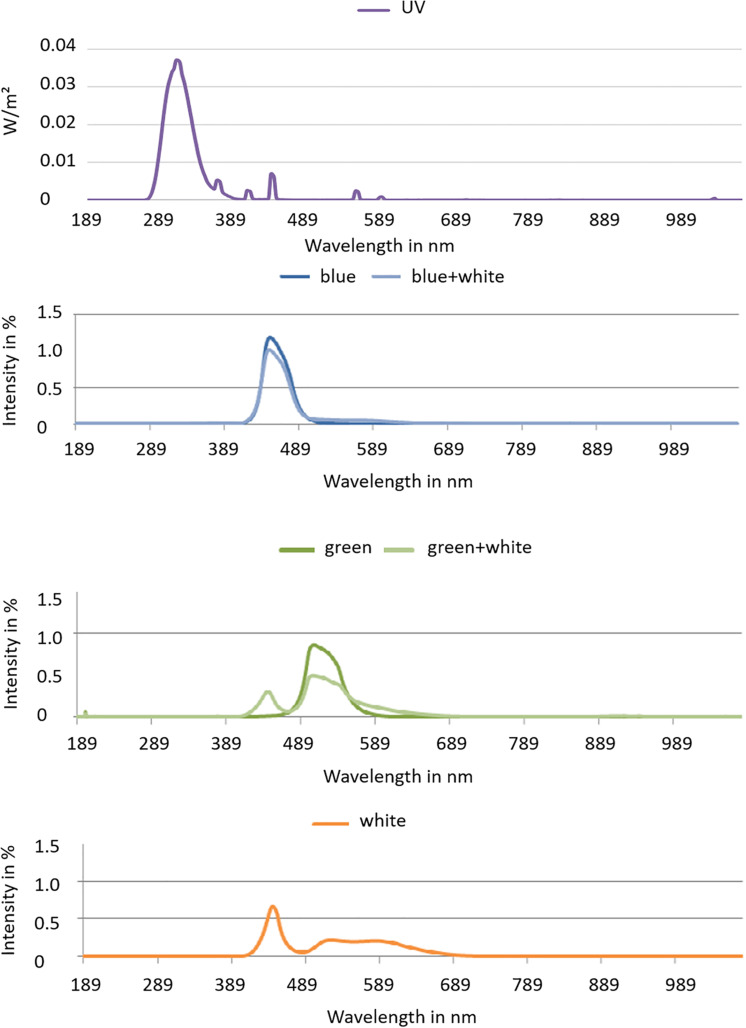
Spectra of blue and green LEDs with and without white light and the white light spectrum used in the climate chambers.

Three Brassicaceae vegetables, namely, kale (*Brassica oleracea* var. *sabellica*) kohlrabi (*Brassica oleracea* var. *gongylodes* L.), and rocket salad (*Diplotaxis tenuifolia*), were cultivated in a controlled environment, in a greenhouse, for the UV treatment. Afterward, they were transferred to climate chamber for the LED treatment. The first part of this experiment was carried out in the greenhouse at Grossbeeren (Germany, 52.37°N 13.33°E) under partly overcast skies with glass that is blocking UV. PAR was on average 306 μmol m^–2^ s^–1^ with intra- and inter-day variations [natural sunlight plus Lamps (SON-T Agro 400)]. Temperature was set at 22°C from 8:00 to 21:00 and 18°C from 21:00 to 8:00. The sprouts (3 days after sowing) were treated with five subsequent doses of moderate UV-B provided by UV-B fluorescence light sources (TL 40W 12 RS, Philips, Hamburg, Germany). The UV-B dose was 0.23 kJ m^–2^ day^–1^ UV-B_BE_ per day (total: 1.15 kJ m^–2^ day^–1^ UV-B_BE_) at a distance of 60 cm from 10:30 to 12:00 each day. The lamps were moving above the samples being applied to an irrigation system, which at the same time results in an balanced UV radiation treatment among the samples. To calculate the biologically effective UV-B, the generalized plant action spectrum (Caldwell, 1971) was used. UV-B radiation was determined using an UV-B sensor (type DK-UVB 1.3-051, deka Sensor + Technologie, Teltow, Germany) with a spectral range of 265–315 nm. Afterward, a subset of the 8-day-old sprouts pre-treated with UV got two subsequent doses (6 h each from 8:00 to 14:00) of blue (450 nm–99 μmol m^–2^ s^–1^) or green (510 nm–119 μmol m^–2^ s^–1^) light (Figure 1). In addition to the blue or green LEDs, white LEDs (400–700 nm) were present in the climate chamber with 100 μmol m^–2^ s^–1^ from 6:00 to 18:00 to ensure further growth of the plants (Figure 1). Temperature was 22°C from 8:00 to 21:00 and 18°C from 21:00 to 8:00. A sampling fraction of three biological replicates, each one tray of sprouts grown from 3 g of seeds, was harvested after an acclimatization period of 24 h after the last treatment.

Flavonoid glycosides and hydroxycinnamic acids were identified and quantified by HPLC-ESI-MS^n^ (Neugart et al., 2012b). The modified method follows in brief, and lyophilized samples (20 mg) were extracted with 1.2 ml of 60% aqueous methanol in three steps. The extract was evaporated to dryness and the residue was dissolved in 200 μl of 10% methanol and then filtered through a Spin-X filter (cellulose acetate membrane filter) for the HPLC analysis. An HPLC series 1100 from Agilent (Waldbronn, Germany) consisting of a degasser, binary pump, autosampler, column oven, and photodiode array detector was used to quantify the flavonoid glycosides. For identification purposes, an ion trap mass spectrometer (Agilent series 1100 MSD) was used with ESI as an ion source in negative ionization mode. Nitrogen was the dry gas (12 L/min, 350°C) and nebulizer gas (40 psi). Helium was the inert collision gas in the ion trap. The following gradient was used for eluent B (100% acetonitrile) at a temperature of 30°C: 5–7% (0–12 min), 7–9% (12–25 min), 9–12% (25–45 min), 12–15% (45–100 min), 15% isocratic (100–150 min), 15–50% (150–155 min), 50% isocratic (155–165 min), 50–5% (165–170 min), 5% isocratic (170–175 min). The flow was performed using 0.4 ml min^–1^, and the measured detector wavelength for the quantification was set at 370 nm for non-acylated flavonol glycosides and 330 nm for acylated flavonol glycosides. The standards quercetin-3-*O*-glucoside and the corresponding 3-*O*-glucosides of kaempferol and isorhamnetin (Carl Roth GmbH, Karlsruhe, Germany) were used to obtain an external calibration curve in the range of 0.1–10 mg 100 ml^–1^.

Singlet oxygen (^1^O_2_) and hydroxyl radical (^∙^OH) scavenging capacities by photospectrometric methods (Majer and Hideg, 2012). In singlet oxygen scavenging capacity measurements, *p*-nitrosodimethylaniline (RNO) is bleached by a product of the reaction between singlet oxygen and histidine, which can be followed by monitoring the decrease in RNO absorption at 440 nm. Reaction mixtures contained 10 mM methylene blue, 15 mM RNO, and 10 mM histidine in 50 mM phosphate buffer (pH 7.0). Singlet oxygen scavenging capacities of plant extracts were measured based on their abilities to inhibit the above reaction and were quantified as millimolar Trolox equivalents per gram of dry matter. The hydroxyl radical scavenging capacity was determined by measuring the ability of leaf extracts to inhibit the formation of the strongly fluorescent 2-hydroxyterephthalate (HTPA) in a reaction between terephthalate (1,4-benzenedicarboxylic acid, TPA) and hydroxyl radical. HTPA fluorescence was measured at room temperature with a Quanta Master QM-1 spectrofluorometer (Photon Technology Inc., Birmingham, NJ, United States), using 315-nm excitation and 420-nm emission. The 2.5-ml reaction mixture contained 500 mM TPA, 10 mM EDTA, 10 mM FeSO_4_, 100 mM AA, and 100 mM H_2_O_2_ in a 50 mM Na-phosphate buffer (pH 7.2). Hydroxyl radical scavenging of each plant extract was characterized by its half-inhibitory concentration on HTPA formation. The method was calibrated with ethanol, which is a strong hydroxyl radical scavenger, and specific hydroxyl radical neutralizing capacities of leaf extracts were given as millimolar ethanol equivalents per gram of dry matter.

The absorption spectra of the extracts were determined according to Mirecki and Teramura (1984). Twenty milligrams of freeze-dried powder was extracted with acidified methanol and used for spectrophotometric determination of total UV-B absorption (A_280__–__315__nm_) which is linked to the phenolic content. All absorption measurements were carried out using a Shimadzu UV-1601 spectrophotometer.

All measurements were done on three independent biological replicates and two technical replicates in the lab.

To analyze variance (ANOVA), Tukey’s Honest Significant Difference (HSD) test was used to calculate significant differences at a significance level of *p* ≤ 0.05.

## Results

### Effect of Light Quality on Structurally Different Flavonoid Glycosides and Hydroxycinnamic Acids

**FIGURE 2:**
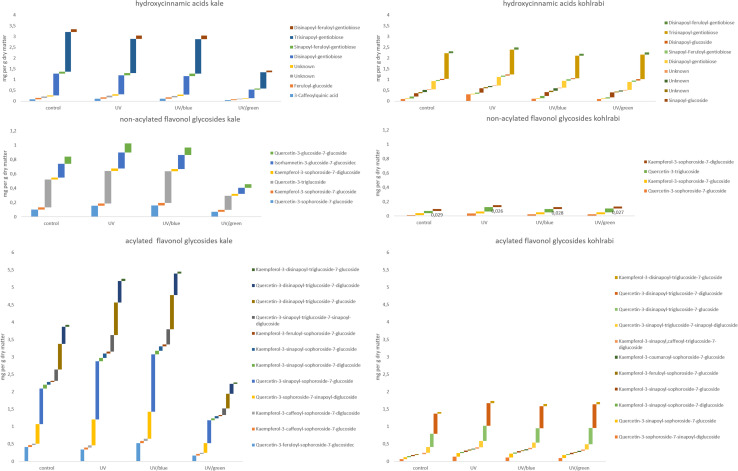
Hydroxycinnamic acids, non-acylated flavonol glycosides, and acylated flavonol glycosides of kale and kohlrabi grown under different irradiation qualities (control, UV, UV plus blue, and UV plus green). For statistics, see Supplementary Tables 1, 2.

**FIGURE 3:**
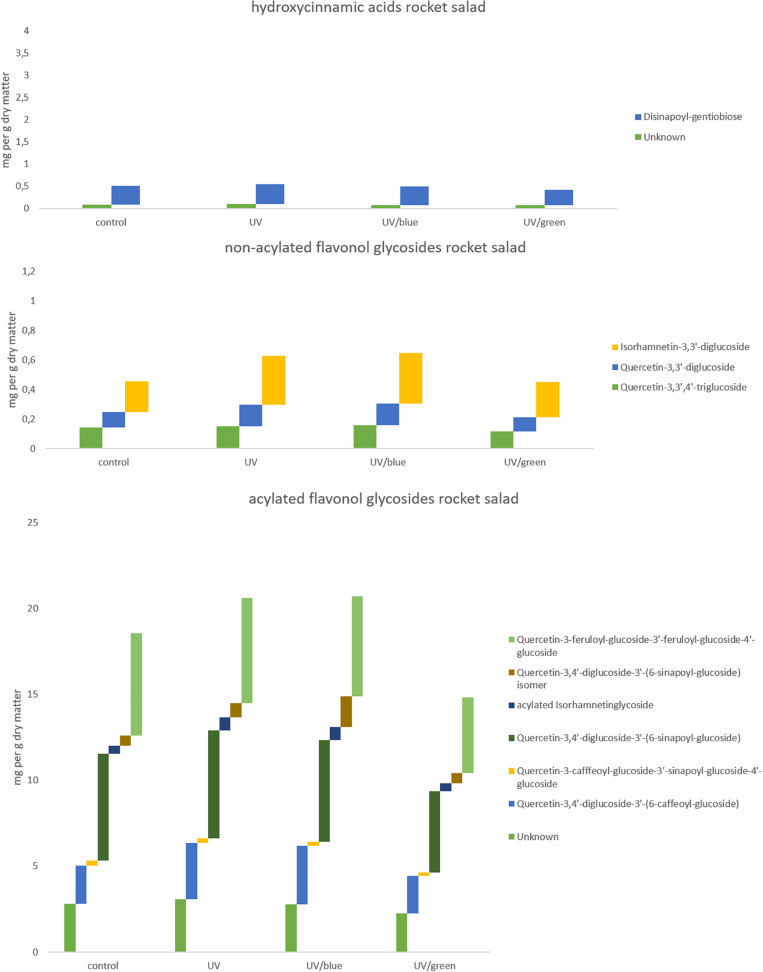
Hydroxycinnamic acids, non-acylated flavonol glycosides, and acylated flavonol glycosides of rocket salad grown under different irradiation qualities (control, UV, UV plus blue, and UV plus green). For statistics see Supplementary Table 3.

The flavonol glycoside and hydroxycinnamic acid profiles varied within the three Brassicaceae vegetables. In total, kale (Figure 2 and Supplementary Table 1) and kohlrabi (Figure 2 and Supplementary Table 2) were characterized by acylated quercetin and kaempferol glycosides based on the 3-*O*-sophoroside-7-*O*-glycosides. Rocket salad (Figure 3 and Supplementary Table 3) had mainly acylated quercetin glycosides based on the quercetin-3,3′,4′-triglucoside. Generally, the response of flavonol glycosides and hydroxycinnamic acids to the different light treatments was distinctly dependent on their chemical structure. In kale, caffeoylquinic acid and feruloyl glucoside were increased by UV-B exposure and remain high after blue light treatment but not after green light treatment (Figure 2 and Supplementary Table 1). Interestingly, gentiobiosides acylated only with sinapoyl residues were found in high concentrations in kale plants, but mixtures of sinapoyl- and feruloyl-acylated gentiobiosides appeared in high concentrations in plants exposed to UV-B and subsequently to blue light treatment. For non-acylated flavonol glycosides, including quercetin, kaempferol, and isorhamnetin glycosides, an increase was found after UV-B exposure and was mostly stable after two doses of blue light but not green light (Figure 2 and Supplementary Table 1). The same was true for most of the acylated quercetin and kaempferol glycosides (Figure 2 and Supplementary Table 1). Nevertheless, for quercetin-3-feruloyl-sphoroside-7-glucoside, kaempferol-3-caffeoyl-sophoroside-7-glucoside, and kaempferol-3-caffeoyl-sophoroside-7-diglucoside, the highest concentrations were found after UV-B exposure followed by blue light treatment. Although kale and kohlrabi showed similar flavonoid and hydroxycinnamic acid profiles, the response of both to treatment was species-specific. In kohlrabi, hydroxycinnamic acids were rarely affected by UV-B exposure (Figure 2 and Supplementary Table 2). However, hydroxycinnamic acid monoglycosides, namely, caffeoyl-glucoside, sinapoyl-glucoside, and disinapoyl-glucoside, were increased after UV-B exposure followed by the exposure to green light. In contrast, mainly non-acylated quercetin glycosides (quercetin-3-sophoroside and quercetin-3-triglucoside) were increased by two- to threefold in response to UV-B (Figure 2 and Supplementary Table 2). Blue as well as green light after the UV-B exposure resulted in a decrease of the non-acylated quercetin glycosides. Furthermore, in kohlrabi, the most acylated quercetin and kaempferol triglycosides and tetraglycosides were increased by UV-B exposure and were still high after two subsequent doses of blue light, but no green light (Figure 2 and Supplementary Table 2). Nevertheless, quercetin and kaempferol pentaglycosides were not affected by UV-B exposure or blue and green light. The values are still high after two subsequent doses of blue light but not with green light instead. In rocket salad, several flavonol glycosides and hydroxycinnamic acids were increased by 1.2- to 2-fold due to the exposure with UV-B, e.g., quercetin-3′,4′-diglucoside-3′-(6-caffeoyl-glucoside) (Figure 3 and Supplementary Table 3). The values are still high after two subsequent doses of blue light, but not with green light instead.

### Effect of Light Quality on ROS Scavenging Capacity

**FIGURE 4:**
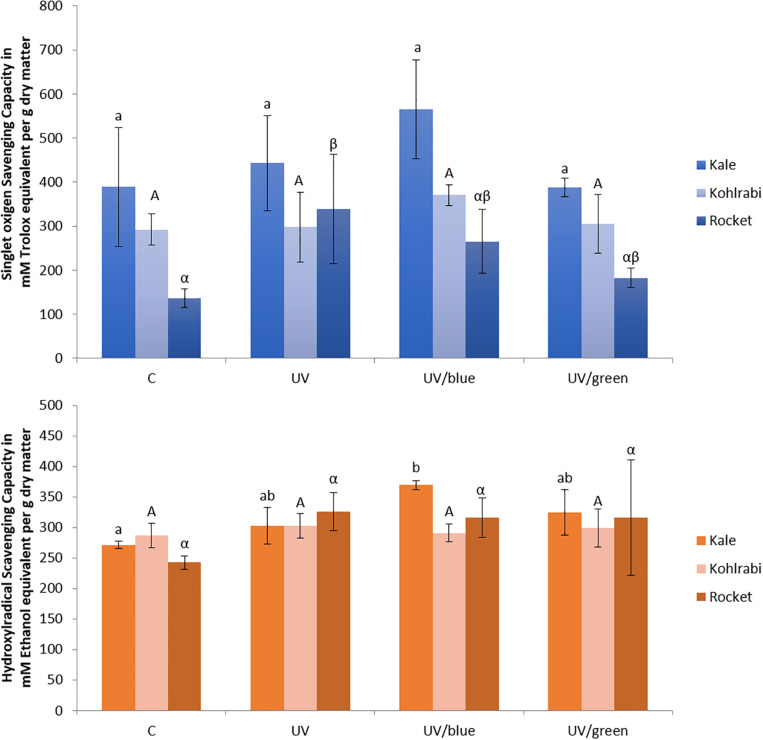
Singlet oxygen scavenging capacity and hydroxyl radical scavenging capacity of three Brassiceae species grown under different irradiation qualities (control, UV plus blue, and UV plus green). Different letters represent significant differences (*p* ≤ 0.05 by Tukey’s HSD test) between the treatment (*n* = 3).

In the present study, kale and kohlrabi, in general, showed a higher singlet oxygen scavenging capacity than rocket salad (Figure 4). Both kale and kohlrabi belong to the *Brassica* genus while rocket salad is *Diplotaxis* species. The results reveal a strong species-specific response of sprouts to the different light qualities regarding singlet oxygen scavenging capacity. For hydroxyl radical scavenging capacity, no species-specific response was detected in the *Brassica* sprouts. UV-B exposure resulted in higher singlet oxygen scavenging capacity in rocket but not kale and kohlrabi (Figure 4). Blue light treatment following the UV-B exposure did not result in a significant increase of singlet oxygen scavenging for kale and rocket but in a trend toward an increase. Green light treatment after UV-B exposure showed similar singlet oxygen scavenging as the control for all three *Brassicaceae* sprouts. The hydroxyl radical scavenging capacity was independent from the light quality except for kale, where an exposure with UV-B followed by a blue light treatment led to a higher hydroxyl radical scavenging capacity. In kale, non-acylated as well as acylated quercetin and kaempferol glycosides of very different chemical structures contributed to the singlet oxygen-scavenging capacity including kaempferol-3-caffeoyl-sophoroside-7-glucoside and its corresponding tetraglycoside (Table 1). In kohlrabi, however, there was not a single compound that contributed significantly to singlet oxygen scavenging capacity (Table 2), whereas in rocket salad, almost all the compounds measured, most of them quercetin glycosides and disinapoyl and trisinapoyl geniobiosides, have contributed to the singlet oxygen scavenging capacity (Table 3). None of the Brassicaceae species studied had a large amount of compounds with hydroxyl radical scavenging capacity (Tables 1–3).

**TABLE 1:** Correlation of hydroxycinnamic acid derivatives and flavonol glycosides (mg g^–1^ dry weight) and biological functions as antioxidants (singlet oxygen scavenging capacity and hydroxyl radical scavenging capacity) and ultraviolet radiation shielding (absorbance) in kale.

	Retention time	Singlet oxygen scavenging capacity	Hydroxyl radical scavenging capacity	Absorbance
**Hydroxycinnamic acid derivatives**				
3-Caffeoylquinic acid	6.2	0.352184	0.051560	–0.061421
Feruloyl-glucosidec	11.6	0.381948	0.081930	–0.087946
Unknown	20.5	0.208944	–0.227761	–0.312001
Unknown	23.5	0.293131	–0.063830	–0.151934
Disinapoyl-gentiobiose	37.0	0.190063	–0.287159	−**0.426992**
Sinapoyl-feruloyl-gentiobiose	37.6	0.385117	0.072049	–0.121618
Trisinapoyl-gentiobiose	44.1	0.204305	–0.273807	−**0.409741**
Disinapoyl-feruloyl-gentiobiose	44.8	0.363088	0.045728	–0.082927
**Non-acylated flavonol glycosides**				
Quercetin-3-sophoroside-7-glucoside	8.6	**0.449189**	0.283069	0.202812
Kaempferol-3-sophoroside-7-glucoside	9.2	0.378105	0.085331	–0.069357
Quercetin-3-triglucoside	9.4	0.332416	–0.011929	–0.120216
Kaempferol-3-sophoroside-7-diglucoside	10.0	**0.458391**	0.327639	0.255956
Isorhamnetin-3-glucoside-7-glucosidec	11.0	0.221753	–0.188276	–0.206147
Quercetin-3-glucoside-7-glucoside	19.8	0.160116	–0.200319	–0.212897
**Acylated flavonol glycosides**				
Quercetin-3-feruloyl-sophoroside-7-glucosidec	12.9	0.383240	0.205756	–0.059238
Kaempferol-3-caffeoyl-sophoroside-7-glucoside	13.2	**0.501781**	0.317331	0.092935
Kaempferol-3-caffeoyl-sophoroside-7-diglucoside	13.5	**0.465506**	**0.452818**	0.365073
Quercetin-3-sophoroside-7-sinapoyl-diglucoside	14.6	0.364968	0.161180	0.057482
Quercetin-3-sinapoyl-sophoroside-7-glucoside	14.8	**0.408538**	0.253602	0.170001
Kaempferol-3-sinapoyl-sophoroside-7-diglucoside	16.1	0.153452	–0.224867	–0.379953
Kaempferol-3-sinapoyl-sophoroside-7-glucoside	16.6	**0.431807**	0.355216	0.297535
Kaempferol-3-feruloyl-sophoroside-7-glucoside	16.9	**0.514344**	**0.552080**	**0.458408**
Quercetin-3-sinapoyl-triglucoside-7-sinapoyl-diglucoside	29.1	0.356813	0.103971	0.036905
Quercetin-3-disinapoyl-triglucoside-7-glucoside	30.3	**0.408943**	0.165905	0.046120
Quercetin-3-disinapoyl-triglucoside-7-diglucoside	30.4	0.390050	0.100338	0.010353
Kaempferol-3-disinapoyl-triglucoside-7-glucoside	31.7	0.345852	0.024576	–0.076607

*Results include three biological replicates each measured as technical duplicates. Bold values indicate significant correlations p ≤ 0.05.*

**TABLE 2:** Correlation of hydroxycinnamic acid derivatives and flavonol glycosides (mg g^–1^ dry weight) and biological functions as antioxidants (singlet oxygen scavenging capacity and hydroxyl radical scavenging capacity) and ultraviolet radiation shielding (absorbance) in kohlrabi.

	Retention time	Singlet oxygen scavenging capacity	Hydroxyl radical scavenging capacity	Absorbance
**Hydroxycinnamic acid derivatives**				
3-Caffeoylquinic acid	6.2	–0.101751	0.137149	0.188011
Caffeoyl-glucoside	6.6	0.300800	0.139749	**0.446213**
Feruloyl-glucoside	11.6	–0.018152	–0.001907	–0.298706
Sinapoyl-glucoside	13.2	0.078954	0.048242	**0.700352**
Unknown	20.6	0.136176	0.244739	–0.158513
Unknown	22.1	–0.031982	0.027816	–0.271488
Unknown	24.2	–0.172506	0.284134	0.042835
Disinapoyl-gentiobiose	37.0	–0.084294	0.145878	0.080536
Sinapoyl-Feruloyl-gentiobiose	37.6	0.154490	0.138082	0.271076
Disinapoyl-glucoside	41.5	–0.091963	–0.038959	0.122176
Trisinapoyl-gentiobiose	44.1	–0.239872	0.206963	–0.092451
Disinapoyl-feruloyl-gentiobiose	44.8	0.072664	0.047670	**0.415865**
**Non-acylated flavonol glycosides**				
Quercetin-3-sophoroside-7-glucoside	8.6	0.202437	0.124999	**0.508333**
Kaempferol-3-sophoroside-7-glucoside	9.2	0.147052	0.097162	0.371887
Quercetin-3-triglucoside	9.4	0.124553	0.022812	**0.569440**
Kaempferol-3-sophoroside-7-diglucoside	10.5	–0.178689	–0.035190	–0.363275
**Acylated flavonol glycosides**				
Quercetin-3-sophoroside-7-sinapoyl-diglucoside	14.5	0.071770	0.034289	**0.490458**
Quercetin-3-sinapoyl-sophoroside-7-glucoside	14.9	0.070899	–0.202322	0.166614
Kaempferol-3-sinapoyl-sophoroside-7-diglucoside	16.1	0.264322	0.061002	0.196169
Kaempferol-3-sinapoyl-sophoroside-7-glucoside	16.6	0.308640	0.102392	**0.491060**
Kaempferol-3-feruloyl-sophoroside-7-glucoside	16.9	0.337465	0.100600	**0.493892**
Kaempferol-3-coumaroyl-sophoroside-7-glucoside	17.2	0.255314	–0.004100	**0.694317**
Kaempferol-3-sinapoyl,caffeoyl-triglucoside-7-diglucoside	28.4	0.006828	–0.023776	–0.211027
Quercetin-3-sinapoyl-triglucoside-7-sinapoyl-diglucoside	29.1	–0.181499	0.012428	0.057980
Quercetin-3-disinapoyl-triglucoside-7-glucoside	30.3	–0.056011	–0.021571	0.375379
Quercetin-3-disinapoyl-triglucoside-7-diglucoside	30.4	–0.035954	–0.005042	0.352710
Kaempferol-3-disinapoyl-triglucoside-7-glucoside	31.7	0.127330	–0.011764	**0.673996**

*Results include three biological replicates each measured as technical duplicates. Bold values indicate significant correlations p ≤ 0.05.*

**TABLE 3:** Correlation of hydroxycinnamin acid derivatives and flavonol glycosides (mg g^–1^ dry weight) and biological functions as antioxidants (singlet oxygen scavenging capacity and hydroxyl radical scavenging capacity) and ultraviolet radiation shielding (absorbance) in rocket salad.

	Retention time	Singlet oxygen scavenging capacity	Hydroxyl radical scavenging capacity	Absorbance
**Hydroxycinnamic acid derivatives**				
Unknown	22.0	**0.662612**	0.151068	0.181309
Disinapoyl-gentiobiose	37.0	**0.596333**	0.027661	0.059041
Trisinapoyl-gentiobiose	44.1	**0.618794**	0.050132	0.132872
**Non-acylated flavonol glycosides**				
Quercetin-3,3′,4′-triglucoside	7.0	**0.522826**	–0.008168	–0.039766
Quercetin-3,3′-diglucoside	19.8	**0.780204**	0.295542	0.095067
Isorhamnetin-3,3′-diglucoside	21.8	**0.748274**	0.402655	0.236553
**Acylated flavonol glycosides**				
Unknown	13.1	**0.596496**	0.078941	0.124960
Quercetin-3,4′-diglucoside-3′-(6-caffeoyl-glucoside)	15.8	**0.782141**	0.338902	0.115805
Quercetin-3-cafffeoyl-glucoside-3′-sinapoyl-glucoside-4′-glucoside	19.3	0.224488	–0.257167	–0.084739
Quercetin-3,4′-diglucoside-3′-(6-sinapoyl-glucoside)	25.9	**0.471698**	–0.084599	–0.037693
Acylated isorhamnetin glycoside	33.4	**0.825667**	**0.428811**	0.089431
Quercetin-3,4′-diglucoside-3′-(6-sinapoyl-glucoside) isomer	33.8	0.194651	–0.001776	0.203347
Quercetin-3-feruloyl-glucoside-3′-feruloyl-glucoside-4′-glucoside	35.2	**0.538342**	–0.027237	–0.012547

*Bold values indicate significant correlations p ≤ 0.05.*

### Effect of Light Quality on Absorption Ability

**FIGURE 5:**
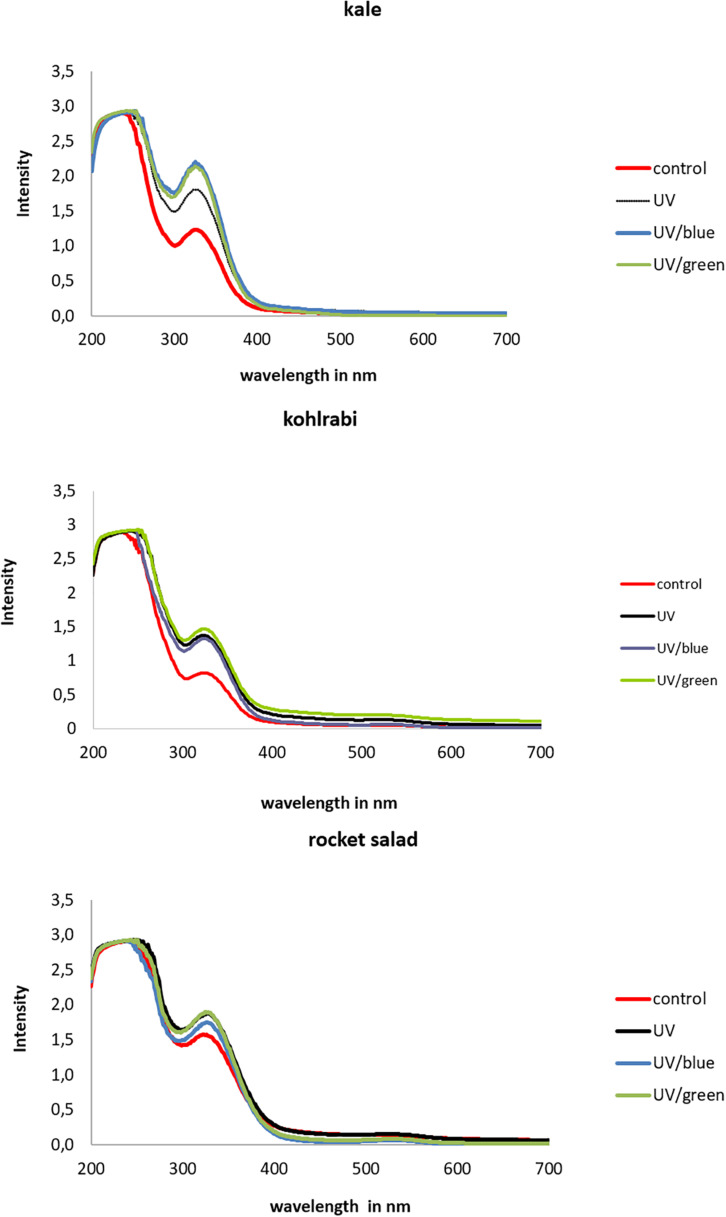
Absorption of three Brassiceae species grown under different irradiation qualities (control, UV, UV plus blue, and UV plus green).

The extracts of Brassicaceae vegetables showed lower absorption intensities in kohlrabi compared to kale and rocket (Figure 5). In kale, the absorption was higher in UV-B-exposed plants and was additionally increased by the blue and green light treatment, whereas in kohlrabi, there was a clear UV-B exposure effect but no further increase following the blue and green light treatment. In rocket salad, the absorption was only slightly increased by UV-B exposure and not further increased by blue or green light treatment. Nevertheless, for kale, negative correlations of absorption and the hydroxycinnamic acid derivatives disinapoyl-gentiobioside as well as trisinapoly gentiobioside were found, whereas kaempferol-3-feruloyl-sophorroside-7-glucoside was the only flavonol glycoside to contribute to the absorption (Table 1). In contrast, in kohlrabi caffeoyl-glucoside, sinapoyl-glucoside and disnapoyl-feruloyl-gentiobioside contributed to the higher absorption alongside with several non-acylated quercetin glycosides and acylated quercetin and kaempferol glycosides (Table 2). In rocket salad, none of the soluble flavonol glycosides and hydroxycinnamic acid derivatives contributed to absorbance changes, which were very small (Table 3).

## Discussion

### Effect of Light Quality on Structurally Different Flavonoid Glycosides and Hydroxycinnamic Acids

The results on the chemical structures identified and quantified are in line with the literature on *Brassica* species (Ferreres et al., 2009; Lin and Harnly, 2009; Olsen et al., 2009; Schmidt et al., 2010; Cartea et al., 2011; Kyriacou et al., 2020) and rocket salad (Martínez-Sánchez et al., 2007; Pasini et al., 2012; Bell and Wagstaff, 2014). UV-B exposure can lead to an up to 2 fold increase of phenolic compounds in *Brassica* species and rocket salad as previously found in the literature (Reifenrath and Mueller, 2007; Harbaum-Piayda et al., 2010; Mormile et al., 2019; Table 4). Additionally, an effect dependent on the specific chemical structure as well as the dose and duration of the UV-B treatment was shown (Olsson et al., 1998; Neugart et al., 2012a, 2014; Groenbaek et al., 2014; Rechner et al., 2016). The present study shows that kaempferol glycosides such as kaempferol-3-caffeoyl-sophoroside-7-glucoside of *Brassica* species and quercetin glycosides in rocket salad are involved in the UV and blue light response. Nevertheless, blue light was not able to further increase flavonoid glycosides and hydroxycinnamic acids in Brassicaceae sprouts. However, in broccoli, kaempferol-3-feruloyl-sphoroside-7-glucoside and kaempferol-3-caffeoyl-sophoroside-7-glucoside were increased after blue light treatment alone, without any UV pre-treatment (Rechner et al., 2017). Blue light was able to increase total phenolics of lettuce (Johkan et al., 2010). Furthermore, in basil, lettuce and arugula flavonoid glycosides and hydroxycinnamic acids reacted to blue light based on their chemical structure, mainly based on the aglycone as trigger (Taulavuori et al., 2016, 2018). An effect of the glycosylation pattern or the acylated hydroxycinnamic acid has not been found in the present study but was previously discussed for kale juvenile plants or broccoli juvenile plants treated with UV-B (Neugart et al., 2014; Rechner et al., 2016). Furthermore, blue light was able to increase sinapoyl derivatives and kaempferol glycosides of *Arabidopsis thaliana* with a clear pattern that kaempferol glycosides including glucose are increased, while those not including glucose but rhamnose are not (Brelsford et al., 2019). The fact that complex flavonoid glycosides, e.g., quercetin and kaempferol pentaglycosides from kohlrabi, are not affected by the light treatment was previously found in broccoli (Rechner et al., 2016). It is worth noting that the blue-to-red ratio should be 1:3 or higher to increase flavonoid glycosides and hydroxycinnamic acids (Pennisi et al., 2019). Such ratios were not reached in the present study and might explain why no further increase of flavonoid glycosides and hydroxycinnamic acid derivatives was found. However, green light seems to reduce plant response in general (Folta and Maruhnich, 2007), even though there had also been reports that green light in addition to blue and red light can increase total phenols in lettuce (Bian et al., 2018). In accordance with the results of the present study in broccoli plants radiated with green light without any treatment of UV-B, structurally different flavonoid glycosides and hydroxycinnamic acids were in the same concentrations of the control plants (Rechner et al., 2017). This is true although green light (119 μmol m^–2^ s^–1^) had a higher intensity than blue light (99 μmol m^–2^ s^–1^) due to technical limitations in the climate chamber. Generally, there is the assumption that higher intensities of blue and green light are needed to actually increase flavonoid glycosides and hydroxycinnamic acid derivatives.

**TABLE 4:** Overview of the effects of UV, blue, and green light on flavonoid glycosides and hydroxycinnamic acid derivatives.

Species	Irradiation treatment	Induced changes in phenolic compounds UV light	References
Pak choi (*Brassica rapa* ssp. *chinensis*)	0.35 W m^–2^ UV-B	Kaempferol glycosides ↑ Malates of hydroxycinnamic acids	Harbaum-Piayda et al., 2010
Rocket salad (Eruca sativa)	No UV vs 60% UV transmission	Quercetin glycosides ↑ Luteoloin glycosides ↑ Apigenin glycosides ↑ Hydroxycinnamic acids ↓-	Mormile et al., 2019
Kale (*Brassica oleracea* var. *sabellica*)	0.22-0.88 kJ m^–2^ day^–1^ UV-B_BE_ (single dose)	Quercetin glycosides ↓ Kaemperol glycosides ↑↓ (depending on glycosylation and acylation pattern)	Neugart et al., 2012b
Kale (*Brassica oleracea* var. *sabellica*)	0.25–1.25 kJ m^–2^ day^–1^ UV-B_BE_	Quercetin glycosides ↑ Kaemperol glycosides ↑↓ (depending on glycosylation and acylation pattern)	Neugart et al., 2014
Canola (*Brassica napus*)	2 kJ m^–2^ day^–1^ UV-B_BE_	Quercetin glycosides ↓ Kaemperol glycosides ↑↓ (depending on glycosylation and acylation pattern)	Olsson et al., 1998
Broccoli (*Brassica oleracea* var. *italica* Plenck)	2.9 kJ m^–2^ day^–1^ UV-B	Quercetin glycosides ↑- Kaempferol glycosides ↑- Hydroxycinnamic acids ↓-	Rechner et al., 2016
Nasturtium (*Nasturtium officinale*), Mustard (*Sinapis alba*)	1.12 W m^–2^ UV-B	Quercetin glycosides ↑ Kaempferol glycosides ↑ Hydroxycinnamic acids -	Reifenrath and Mueller, 2007

**Species**	**Irradiation treatment**	**Induced changes in phenolic compounds blue light**	**References**

*Arabidopsis thaliana*	PAR 168 μmol m^–2^ s^–1^ plus 32 μmol m^–2^ s^–1^ far red, blue light attenuated by film	Kaempferol glycosides ↑- Hydroxycinnamic acids ↑	Brelsford et al., 2019
Red leaf lettuce (*Lactuca sativa* L.)	100 μmol m^–2^ s^–1^	Total phenols ↑ Antocyanins ↑	Johkan et al., 2010
Chinese kale (*Brassica alboglabra*)	0–150 μmol m^–2^ s^–1^	Total phenols ↑ Antocyanins ↑	Li et al., 2019
Broccoli (*Brassica oleracea* var. *italica* Plenck)	50 μmol m^–2^ s^–1^ blue light	Quercetin glycosides - Kaempferol glycosides ↑- Hydroxycinnamic acids -	Rechner et al., 2017
Red leaf lettuce (*Lactuca sativa*) Basil (*Ocimum basilicum*)	300 μmol m^–2^ s^–1^ (blue enhanced)	Quercetin glycosides ↑- Hydroxycinnamic acids ↑-	Taulavuori et al., 2016
Basil (*Ocimum basilicum*), arugula (*Eruca sativa*), and bloody dock (*Rumex sanguineus*)	300 μmol m^–2^ s^–1^ (blue enhanced)	Apigenin glycosides ↑- Luteolin glycosides ↑- Hydroxycinnamic acids ↑-	Taulavuori et al., 2018
Lamb’s lettuce (*Valerianella locusta*)	200 μmol m^–2^ s^–1^ 50% red and 50% blue, 70% red and 30% blue, 90% red and 10% blue	Total phenols ↓	Wojciechowska et al., 2015
Pak choi (*Brassica rapa* ssp. *chinensis*)	0–150 μmol m^–2^ s^–1^	Total phenols ↑ Antocyanins ↑	Zheng et al. (2018)

**Species**	**Irradiation treatment**	**Induced changes in phenolic compounds green light**	**References**

Lettuce (*Lactuca sativa* L.)	Red:blue:green = 1:1:1 150 μmol m^–2^ s^–1^	Total phenols ↑	Bian et al., 2018
Broccoli (*Brassica oleracea* var. *italica* Plenck)	50 μmol m^–2^ s^–1^ blue light	Quercetin glycosides - Kaempferol glycosides - Hydroxycinnamic acids -	Rechner et al., 2017

Even though kale and kohlrabi had similar flavonoid and hydroxycinnamic acid profiles, the response of both to the treatment was species-specific (Figure 1). In Chinese kale, 100 μmol m^–2^ s^–1^ of blue light treatment were not enough to increase total phenolics (Li et al., 2019), while in pak choi, it was enough to lead to an increase of total phenolics (Zheng et al., 2018). In lamb’s lettuce, the increase of blue light in the light spectrum even decreased the total phenolics (Wojciechowska et al., 2015). It is therefore possible that either the intensity of blue light was too low for kohlrabi or kohlrabi uses other defense mechanisms such as waxes.

### Effect of Light Quality on ROS Scavenging Capacity

Leaves are good sources of flavonoid glycosides, which, alongside with other compounds such as carotenoids and vitamins, contribute to a high singlet oxygen scavenging capacity (Ribeiro et al., 2015). Ascorbic acid and quercetin contribute to the hydroxyl radical scavenging capacity of Indian olive (Panja et al., 2014). This leads to the conclusion that other compounds must be involved. In the present study, the results reveal a strong species-specific response of sprouts to the different light qualities dependent on the high variation of flavonoid glycosides and hydroxycinnamic acids within the species. Recent results showed a strong correlation between flavonols and singlet oxygen scavenging capacity, as shown here for kale and rocket salad, but also revealed that glycosides of flavonols are less effective as their aglycones (Agati et al., 2007; Majer et al., 2014; Csepregi and Hideg, 2018). Hydroxycinnamic acids had about 10% of singlet oxygen scavenging capacity compared to quercetin and kaempferol (Csepregi and Hideg, 2018). In the Brassicaceae sprouts, no correlation of hydroxycinnamic acids and the singlet oxygen scavenging capacity and hydroxyl radical scavenging capacity was found as previously described for hydroxycinnamic acids ferulic acid and *p-*coumaric acid of millet cultivars (Chandrasekara and Shahidi, 2011). In wild strawberries, different cultivars showed a strong correlation of singlet oxygen scavenging capacity and hydroxyl radical scavenging capacity (Wang et al., 2007), a trend that is also seen in the data presented here. Furthermore, in strawberries, numerous hydroxycinnamic acids as well as quercetin and kaempferol glycosides were increased with higher temperatures, in line with the singlet oxygen scavenging capacity and hydroxyl radical scavenging capacity (Wang and Zheng, 2001). None of the Brassicaceae species studied showed a large amount of compounds contributing to the hydroxyl radical scavenging capacity. Concomitantly, Majer et al. (2010) found no correlation between total phenolic content and hydroxyl radical scavenging capacity in tobacco leaves assuming other compounds involved in the quenching of hydroxyl radical. Hydroxycinnamic acids, although highly concentrated in the *Brassica* sprouts (Heinze et al., 2018), had no specific effects on the scavenging capacity to plant-specific ROS.

### Effect of Light Quality on Absorption Ability

In addition to their role as antioxidants, flavonoids act as UV shielding components (Edreva, 2005). Kaempferol, quercetin, and isorhamnetin absorb longer wavelength in the UV radiation (350–400 nm) better than higher energized UV-B (280–315 nm). Absorption spectra of flavonol glycosides are shifted slightly toward shorter wavelengths compared to their corresponding aglycones (Majer et al., 2014). However, the role of structurally different flavonol glycosides in the UV shielding function of leaves is still not clear. UV-B as well as blue and green light treatment resulted in a slight shift of the band I assuming a better shielding activity (Edreva, 2005). Hydroxycinnamic acids with absorption maxima between 320 and 330 nm serve specific UV-B screening functions much better (Harborne and Williams, 2000). The results on kale and kohlrabi, once more underlines the species-specific response although the compounds were comparable in the two *Brassica oleracea* species. In the model plant *Arabidopsis thaliana*, the absorption at 330 nm was higher in a UV-tolerant mutant, in line with the reduced UV-B transmittance (Bieza and Lois, 2001). Kohlrabi, as a plant, might use also other defense mechanisms such as leaf hairs or waxes. In general, hydroxycinnamic acids have a higher UV-A and especially UV-B absorption compared to the flavonoid aglycones quercetin and kaempferol (Csepregi and Hideg, 2018). However, it is to be mentioned that glycosylation of flavonoid aglycones, which is commonly found in plants, resulted in a lower UV absorption capacity of these compounds (Csepregi and Hideg, 2018). Furthermore, in the absence of flavonoids, hydroxycinnamic acid derivatives were found to take over the absorption of UV radiation especially in the UV-A range (Stelzner et al., 2019). In total, the absorbance seemed to be less important in the defense against UV compared to the antioxidant activity as previously shown in linden leaves (Majer et al., 2014). Nevertheless, cell wall-bound phenolics were not investigated here and might play a major role in the UV response based on absorption.

## Conclusion

UV radiation resulted in the enhancement of a number of hydroxycinnamic acids and quercetin as well as kaempferol glycosides in different *Brassicaceae* vegetable sprouts. In kale, the glycosylation pattern had no effect on the UV-B response. In kohlrabi, tri- and tetraglycosides are involved in the UV-B response, whereas pentaglycosides are not, which is clearly highlighting the effect of the glycosylation pattern. Blue light treatment after pre-exposure with UV resulted in constantly high concentrations of the most hydroxycinnamic acids and quercetin and kaempferol glycosides in three *Brassicaceae* sprouts, but no further increase. Green light, in general, resulted in a decrease of most flavonol glycosides and hydroxycinnamic acids to the control level. This result underlines the importance of different light qualities on the biosynthesis of flavonoids and hydroxycinnamic acids and their fast response to changing environments. The enhancement of the singlet oxygen scavenging capacity or the hydroxyl radical scavenging capacity was very low and often only a trend even though some hydroxycinnamic acids or quercetin and kaempferol glycosides were increased 1.2- to 2-fold. However, sprouts seem to be protected by high concentrations of hydroxycinnamic acids compared to adult plants. The shift in the absorption band highlights the role of flavonol glycosides and hydroxycinnamic acids as shielding compounds in the UV-A and UV-B range. Nevertheless, the investigated sprouts showed species-specific responses in the absorption also reflecting the chemically different structures of the hydroxycinnamic acids and quercetin and kaempferol glycosides, as well as other possible defense mechanisms. Regarding the use of UV light and blue or green light in vertical farming to improve secondary plant metabolites that correlate with quality traits, a combination of UV light and subsequent blue light is possible, but requires further specifications, especially regarding the intensity of blue light.

## Data Availability Statement

The original contributions presented in the study are included in the article/Supplementary Material, further inquiries can be directed to the corresponding author/s.

## Author Contributions

SN and MS designed the experiment. SN conducted the experiment and wrote the first draft of the manuscript. PM measured and evaluated the singlet oxygen and hydroxyl radical scavenging capacity as well as absorbance. ÉH contributed in the discussion of biological functions. All authors revised and approved the manuscript.

## Conflict of Interest

The authors declare that the research was conducted in the absence of any commercial or financial relationships that could be construed as a potential conflict of interest.
