# Supplementary material for: Blue Light Treatment but Not Green Light Treatment After Pre-exposure to UV-B Stabilizes Flavonoid Glycoside Changes and Corresponding Biological Effects in Three Different Brassicaceae Sprouts
Source: Front Plant Sci. 2021 Jan 28;11:611247. doi: 10.3389/fpls.2020.611247 (PMC7875886; doi:10.3389/fpls.2020.611247)
Supplement: Supplementary file 1 [file Data_Sheet_1.PDF]

Table S1 Influence of Ultraviolet (UV)-Radiation followed by blue or green light treatment on flavonol glycosides (mg g^{-1} dry weight) in kale. Results include 3 biological replicates each measured as technical duplicates.

	retention time	control		UV		UV/blue		UV/green	
**Hydroxycinnamic acids**									
3-Caffeoylquinic acid	6,2	0,093±0,003	b	0,113±0,011	c	0,112±0,007	c	0,050±0,013	a
Feruloyl-glucosidec	11,6	0,055±0,002	b	0,064±0,006	c	0,066±0,006	c	0,031±0,006	a
Unknown	20,5	0,067±0,003	b	0,074±0,010	b	0,064±0,006	b	0,031±0,009	a
Unknown	23,5	0,055±0,002	b	0,065±0,007	c	0,061±0,006	bc	0,027±0,007	a
Disinapoyl-gentiobiose	37,0	1,005±0,046	c	0,889±0,045	b	0,865±0,055	b	0,398±0,131	a
Sinapoyl-feruloyl-gentiobiose	37,6	0,101±0,004	b	0,105±0,006	b	0,118±0,010	c	0,049±0,012	a
Trisinapoyl-gentiobiose	44,1	1,835±0,093	c	1,590±0,094	b	1,602±0,084	b	0,754±0,248	a
Disinapoyl-feruloyl-gentiobiose	44,8	0,135±0,005	b	0,150±0,009	bc	0,157±0,010	c	0,075±0,020	a
**non-acylated Flavonolglycosides**									
Quercetin-3-sophoroside-7-glucoside	8,6	0,101±0,004	b	0,154±0,014	c	0,159±0,018	c	0,069±0,018	a
Kaempferol-3-sophoroside-7-glucoside	9,2	0,033±0,000	b	0,033±0,001	bc	0,034±0,001	c	0,028±0,002	a
Quercetin-3-triglucoside	9,4	0,387±0,016	b	0,455±0,036	c	0,443±0,038	bc	0,196±0,056	a
Kaempferol-3-sophoroside-7-diglucoside	10,0	0,027±0,000	a	0,032±0,001	b	0,032±0,001	b	0,026±0,001	a
Isorhamnetin-3-glucoside-7-glucosidec	11,0	0,191±0,011	b	0,223±0,029	c	0,196±0,015	bc	0,088±0,026	a
Quercetin-3-glucoside-7-glucoside	19,8	0,103±0,004	b	0,128±0,017	c	0,105±0,008	b	0,050±0,014	a
**acylated Flavonolglycosides**									
Quercetin-3-feruloyl-sophoroside-7-glucosidec	12,9	0,413±0,030	b	0,341±0,044	b	0,520±0,086	c	0,168±0,038	a
Kaempferol-3-caffeoyl-sophoroside-7-glucoside	13,2	0,055±0,002	b	0,056±0,007	b	0,067±0,005	c	0,043±0,005	a
Kaempferol-3-caffeoyl-sophoroside-7-diglucoside	13,5	0,036±0,001	b	0,059±0,004	b	0,061±0,007	c	0,035±0,002	a
Quercetin-3-sophoroside-7-sinapoyl-diglucoside	14,6	0,566±0,028	a	0,746±0,142	b	0,775±0,068	b	0,275±0,102	a
Quercetin-3-sinapoyl-sophoroside-7-glucoside	14,8	1,024±0,057	b	1,673±0,150	c	1,653±0,209	c	0,658±0,168	a

Kaempferol-3-sinapoyl-sophoroside-7-diglucoside	16,1	0,112±0,004	b	0,098±0,007	c	0,103±0,005	c	0,057±0,011	a
Kaempferol-3-sinapoyl-sophoroside-7-glucoside	16,6	0,075±0,003	c	0,129±0,008	b	0,127±0,012	bc	0,065±0,011	a
Kaempferol-3-feruloyl-sophoroside-7-glucoside	16,9	0,030±0,001	a	0,048±0,002	b	0,050±0,005	b	0,032±0,002	a
Quercetin-3-sinapoyl-triglucoside-7-sinapoyl-diglucoside	29,1	0,328±0,011	b	0,476±0,047	b	0,438±0,036	b	0,190±0,065	a
Quercetin-3-disinapoyl-triglucoside-7-glucoside	30,3	0,736±0,054	b	0,934±0,079	c	0,981±0,090	c	0,420±0,131	a
Quercetin-3-disinapoyl-triglucoside-7-diglucoside	30,4	0,493±0,036	b	0,625±0,053	c	0,619±0,050	c	0,282±0,087	a
Kaempferol-3-disinapoyl-triglucoside-7-glucoside	31,7	0,054±0,002	b	0,058±0,002	b	0,059±0,003	b	0,040±0,006	a
