# Supplementary material for: Blue Light Treatment but Not Green Light Treatment After Pre-exposure to UV-B Stabilizes Flavonoid Glycoside Changes and Corresponding Biological Effects in Three Different Brassicaceae Sprouts
Source: Front Plant Sci. 2021 Jan 28;11:611247. doi: 10.3389/fpls.2020.611247 (PMC7875886; doi:10.3389/fpls.2020.611247)
Supplement: Supplementary file 2 [file Data_Sheet_2.PDF]

Table S2 Influence of Ultraviolet (UV)-Radiation followed by blue or green light treatment on flavonol glycosides (mg g^{-1} dry weight) in kohlrabi. Results include 3 biological replicates each measured as technical duplicates.

	retention time	control		UV		UV/blue		UV/green	
**Hydroxycinnamic acids**									
3-Caffeoylquinic acid	6,2	0,106±0,009	a	0,313±0,458	a	0,113±0,013	a	0,097±0,006	a
Caffeoyl-glucoside	6,6	0,023±0,002	a	0,025±0,001	ab	0,025±0,002	ab	0,026±0,001	b
Feruloyl-glucoside	11,6	0,091±0,051	a	0,051±0,002	a	0,100±0,065	a	0,060±0,006	a
Sinapoyl-glucoside	13,2	0,147±0,027	a	0,210±0,009	bc	0,187±0,031	b	0,219±0,013	c
Unknown	20,6	0,054±0,004	a	0,052±0,003	a	0,056±0,011	a	0,050±0,006	a
Unknown	22,1	0,098±0,077	a	0,048±0,006	a	0,118±0,116	a	0,046±0,010	a
Unknown	24,2	0,030±0,006	ab	0,031±0,001	b	0,023±0,005	a	0,030±0,006	b
Disinapoyl-gentiobiose	37,0	0,380±0,082	a	0,392±0,047	a	0,330±0,066	a	0,370±0,036	a
Sinapoyl-Feruloyl-gentiobiose	37,6	0,059±0,012	a	0,067±0,012	a	0,067±0,012	a	0,063±0,004	a
Disinapoyl-glucoside	41,5	0,053±0,010	ab	0,055±0,002	ab	0,045±0,003	a	0,057±0,010	b
Trisinapoyl-gentiobiose	44,1	1,184±0,080	b	1,154±0,055	b	1,045±0,078	a	1,150±0,079	ab
Disinapoyl-feruloyl-gentiobiose	44,8	0,093±0,008	a	0,100±0,012	a	0,099±0,012	a	0,105±0,005	a
**non-acylated Flavonolglycosides**									
Quercetin-3-sophoroside-7-glucoside	8,6	0,011±0,005	a	0,033±0,004	c	0,022±0,007	b	0,022±0,002	b
Kaempferol-3-sophoroside-7-glucoside	9,2	0,028±0,002	a	0,029±0,001	a	0,028±0,001	a	0,029±0,000	a
Quercetin-3-triglucoside	9,4	0,029±0,008	a	0,063±0,008	c	0,047±0,014	b	0,053±0,006	bc
Kaempferol-3-sophoroside-7-diglucoside	10,5	0,029±0,001	b	0,026±0,001	a	0,028±0,004	ab	0,027±0,000	ab
**acylated Flavonolglycosides**									
Quercetin-3-sophoroside-7-sinapoyl-diglucoside	14,5	0,064±0,012	a	0,132±0,016	c	0,114±0,019	bc	0,099±0,008	b
Quercetin-3-sinapoyl-sophoroside-7-glucoside	14,9	0,059±0,029	a	0,108±0,021	b	0,110±0,045	b	0,081±0,026	ab
Kaempferol-3-sinapoyl-sophoroside-7-diglucoside	16,1	0,028±0,001	a	0,030±0,001	a	0,029±0,001	a	0,029±0,001	a
Kaempferol-3-sinapoyl-sophoroside-7-glucoside	16,6	0,030±0,001	a	0,038±0,002	c	0,037±0,001	c	0,035±0,001	b

Kaempferol-3-feruloyl-sophoroside-7-glucoside	16,9	0,026±0,000	a	0,030±0,001	c	0,030±0,001	c	0,029±0,000	b
Kaempferol-3-coumaroyl-sophoroside-7-glucoside	17,2	0,000±0,000	a	0,027±0,001	c	0,027±0,000	c	0,026±0,000	b
Kaempferol-3-sinapoyl,caffeoyl-triglucoside-7-diglucoside	28,4	0,042±0,003	a	0,039±0,004	a	0,041±0,002	a	0,041±0,003	a
Quercetin-3-sinapoyl-triglucoside-7-sinapoyl-diglucoside	29,1	0,161±0,027	a	0,185±0,030	a	0,150±0,027	a	0,154±0,020	a
Quercetin-3-disinapoyl-triglucoside-7-glucoside	30,3	0,383±0,046	a	0,433±0,114	a	0,416±0,064	a	0,461±0,041	a
Quercetin-3-disinapoyl-triglucoside-7-diglucoside	30,4	0,576±0,070	a	0,649±0,173	a	0,632±0,118	a	0,688±0,061	a
Kaempferol-3-disinapoyl-triglucoside-7-glucoside	31,7	0,050±0,003	a	0,061±0,003	b	0,059±0,003	b	0,062±0,002	b
