# Supplementary material for: Blue Light Treatment but Not Green Light Treatment After Pre-exposure to UV-B Stabilizes Flavonoid Glycoside Changes and Corresponding Biological Effects in Three Different Brassicaceae Sprouts
Source: Front Plant Sci. 2021 Jan 28;11:611247. doi: 10.3389/fpls.2020.611247 (PMC7875886; doi:10.3389/fpls.2020.611247)
Supplement: Supplementary file 3 [file Data_Sheet_3.PDF]

Table S3 Influence of Ultraviolet (UV)-Radiation followed by blue or green light treatment on flavonol glycosides (mg g^{-1} dry weight) in rocket salad. Results include 3 biological replicates each measured as technical duplicates.

	**retention time**	**control**		**UV**		**UV/blue**		**UV/green**	
**Hydroxycinnamic acids**									
Unknown	22,0	0,085±0,011	a	0,099±0,038	a	0,080±0,020	a	0,077±0,014	a
Disinapoyl-gentiobiose	37,0	0,419±0,059	ab	0,450±0,091	b	0,422±0,043	ab	0,339±0,027	a
Trisinapoyl-gentiobiose	44,1	0,359±0,053	ab	0,393±0,093	b	0,344±0,038	ab	0,289±0,024	a
**non-acylated Flavonolglycosides**									
Quercetin-3,3',4'-triglucoside	7,0	0,142±0,018	ab	0,151±0,025	b	0,157±0,021	b	0,115±0,013	a
Quercetin-3,3'-diglucoside	19,8	0,107±0,012	a	0,148±0,030	b	0,146±0,016	b	0,099±0,009	a
Isorhamnetin-3,3'-diglucoside	21,8	0,206±0,038	a	0,331±0,044	b	0,346±0,032	b	0,236±0,047	a
**acylated Flavonolglycosides**									
Unknown	13,1	2,819±0,610	ab	3,064±0,728	b	2,776±0,202	ab	2,252±0,236	a
Quercetin-3,4'-diglucoside-3'-(6-caffeoyl-glucoside)	15,8	2,225±0,239	a	3,303±0,654	b	3,399±0,369	b	2,193±0,188	a
Quercetin-3-cafffeoyl-glucoside-3'-sinapoyl-glucoside-4'-glucoside	19,3	0,290±0,044	b	0,257±0,060	ab	0,248±0,038	ab	0,202±0,016	a
Quercetin-3,4'-diglucoside-3'-(6-sinapoyl-glucoside)	25,9	6,229±0,739	b	6,298±1,265	b	5,906±0,578	b	4,726±0,343	a
acylated Isorhamnetinglycoside	33,4	0,443±0,119	a	0,737±0,236	b	0,764±0,026	b	0,470±0,073	a
Quercetin-3,4'-diglucoside-3'-(6-sinapoyl-glucoside) isomer	33,8	0,610±0,074	a	0,848±0,158	a	1,808±2,078	a	0,567±0,063	a
Quercetin-3-feruloyl-glucoside-3'-feruloyl-glucoside-4'-glucoside	35,2	5,951±0,744	b	6,112±1,392	b	5,799±0,488	b	4,423±0,394	a

Different letters represent significant differences at $p \leq 0.05$.
